# Tailored Therapy Based on Molecular Characteristics in Endometrial Cancer

**DOI:** 10.1155/2021/2068023

**Published:** 2021-05-05

**Authors:** Suk-young Lee

**Affiliations:** Division of Hemato-Oncology, Department of Internal Medicine, School of Medicine, Wonkwang University, 321 Sanbonro, Gunpo, 15865 Gyeonggi-do, Republic of Korea

## Abstract

Management of endometrial cancer, an adenocarcinoma of the endometrium which occupies most uterine corpus neoplasms, including uterine sarcomas, has been more relevant due to its increasing incidence. Extensive research on tumorigenesis molecular mechanisms and molecular characterization across cancers has brought paradigm shifts in the treatment of various malignant tumors. Endometrial cancer treatment has been traditionally guided according to the disease extent or histology types, while recent studies on molecular features have led to the introduction of targeted agents into clinical use, along with conventional chemotherapeutic agents in patients with recurrent or metastatic disease. Considering the proven efficacy and relatively tolerable toxicities of targeted therapies across malignant tumors, improvement of treatment outcomes is also expected in endometrial cancer by adopting an individualized therapy depending on the specific molecular features. Efficacy assessment of new biological agents is still ongoing based on previous preclinical data on endometrial cancer molecular features. Here, endometrial cancer molecular characterization will be reviewed, and then, we will introduce preclinical data, directing the adoption of new biological agents.

## 1. Introduction

Endometrial cancer (EC), arising from the epithelium of the uterine corpus, is one of the leading causes of cancer mortality among women in developed countries [1, 2]. EC has been classically classified into two types, according to the biological behavior and prognosis. Type I EC arises in patients with obesity, hyperlipidemia, and hyperestrogenism, showing a well to moderately differentiated histology of endometrioid tumors. Meanwhile, type II ECs are absent of the above features, showing a poorly differentiated histology, mostly comprised of serous carcinoma, clear cell carcinoma, or carcinosarcoma. Clinically aggressive features are represented by an advanced stage at diagnosis, a tendency for deep invasion into the myometrium, or frequent recurrence leading to a poor prognosis [3]. Current strategies in EC treatment are mainly guided by histological features and extent of disease. However, although patients with EC in advanced stages or a high-grade pathology are recommended to receive adjuvant chemotherapy, the role of adjuvant systemic chemotherapy is still controversial in terms of overall survival, and an optimal form of adjuvant therapy has still not been established. In addition, data on efficacy of systemic chemotherapy in the treatment of patients with recurrent or metastatic ECs is also limited [4, 5].

Along with tremendous efforts on clarifying the molecular mechanisms of tumorigenesis across cancers, therapies targeting molecules involved in carcinogenesis have been dramatically developed for several decades. Having been fueled by successful treatment outcomes of targeted therapies, guiding treatment of malignant tumors according to molecular aberrations is now generally regarded as one of treatment options for promising outcomes. On the other hand, EC has been relatively distant from benefiting from targeted therapies compared to other malignant tumors, but recent efforts to understand the disease biology have released results of preclinical studies, leading to the development of clinical trials to test the potential of novel biological agents in EC treatment. In 2016, the U.S. National Cancer Institute (NCI) organized a Uterine Clinical Trial Planning Meeting to promote the design of clinical trials for the advance of molecular targeted therapies in EC [6]. In addition to known efficacy of the immunotherapy in patients with mismatch repair- (MMR-) deficient EC, opportunities to examine the potential for new biological agents were given by holding this meeting, and results of clinical trials have been gradually released.

Here, we reviewed the molecular characterization and preclinical studies connected to molecular-targeted therapies in ECs. We also introduce results of clinical trials on novel biological agents and suggest future directions to solve current limitations.

## 2. Preclinical Data Directing Molecular Targeted Therapy

### 2.1. Endometrioid EC

Somatic mutation of *PTEN*, involved in the phosphoinositide-3-kinase (PI3K)/Akt/mammalian target of rapamycin (mTOR) signaling, has been known to be a molecular aberration commonly found in endometrioid EC [5, 7]. The development from normal glandular cells to endometrioid EC is dynamic and complex and involves accumulation of numerous genetic changes, besides stimulation of endometrial epithelium by estrogen [7–9]. Loss of function (LOF) mutation of *PTEN* is an early event occurring in normal glandular cells, but it is insufficient to initiate tumorigenesis of endometrioid EC [10, 11]. Numerous studies have reported that the cooccurrence of mutations in *PTEN* and other genes, such as the activating mutation of *PIK3CA* (catalytic subunit *α* of PI3K), *PIK3R1*, *PIK3R2*, *MLH1* inactivation, LOF mutation of *ARID1A*, and mutation of *CTNNB1*, contributed to endometrial carcinogenesis [11–18].

The role of *PIK3CA* mutation on the existing *PTEN* mutation in complex atypical hyperplasia of endometrial epithelium has been studied. Knockdown of PTEN expression in a cell line with a *PIK3CA* mutation resulted in the enhancement of phosphorylation of Akt, suggesting that an aberration of PIK3CA exerts an additive effect on PI3K activation, leading to endometrioid carcinogenesis [15]. The cooperative role of *PIK3CA* mutation with PTEN inactivation in endometrioid carcinogenesis was also investigated with genetically engineered mouse models. The endometrial epithelium of mouse models harboring *PIK3CA*^E545K^ developed into hyperplasia or cancer, while mice models with an activated mutation of *PIK3CA* on the underlying *PTEN* loss caused endometrial carcinoma, suggesting the distinct but integrative role of PTEN inactivation and activated PIK3CA [11]. The importance of additional genetic aberration on the background *PTEN* mutation has also been demonstrated using heterozygous *PTEN* and biallelic *MLH1*-deficient (*PTEN^+/-^MLH^−/−^*) mice, by showing an acceleration of endometrial tumorigenesis [16]. The role of concurrent mutations of multiple driver genes in endometrioid carcinogenesis was also shown in an *in vivo* castrated female mouse model harboring a *CTNNB1* exon 3 mutation, a LOF mutation of *PTEN*, and a *PIK3CA* activating mutation in the endometrial epithelium. A *CTNNB1* mutation was revealed to be critical in the growth of ovarian steroids retaining preneoplastic epithelial cells with *PTEN* and *PIK3CA* mutations and myometrial invasion of endometrioid carcinoma in castrated mice harboring *PTEN* LOF and *PIK3CA* activating mutations [19]. The *ARID1A* tumor suppressor gene, a commonly found mutation across cancers, has also showed its potential in endometrial carcinogenesis by integrating with PTEN inactivation. Immunohistochemical staining to determine the expression of ARID1A and PTEN in endometrial carcinoma revealed a concurrent occurrence of ARID1A and PTEN inactivation. Proliferative activity was shown to be significantly increased in areas with a concurrent loss of PTEN and ARID1A expression, compared to areas with PTEN loss alone. An *in vitro* cell culture assay also showed a greater proliferative activity in human endometrial epithelial cells with concurrent ARID1A and PTEN inactivation than cells with either PTEN or ARID1A inactivated, suggesting the potential of ARID1A as a gatekeeper in the preneoplastic endometrial epithelium to carcinoma transition [18]. Intriguingly, *ARID1A* is known to function as a regulator of the DNA damage checkpoint. A previous study reported that ARID1A is recruited to DNA double-strand breaks (DSBs) via interaction with ATR and facilitates effective DNA DSB end resection, leading to DSB repair through homologous recombination pathways. Additionally, ARID1A deficiency was revealed to contribute to sensitization of cancer cells to Poly (ADP-Ribose) Polymerase (PARP) inhibitors [20].

### 2.2. Serous EC

ECs in this category are generally independent from estrogen. Like endometrioid carcinoma, which develops from a normal endometrial glandular epithelium to endometrial hyperplasia by stimulation of estrogen, subsequently progressing to complex atypical hyperplasia with accumulation of numerous genetic aberrations, serous ECs are also preceded by serous endometrial intraepithelial carcinoma (SEIC), which evolves from an atrophic endometrial glandular epithelium [7, 8, 21]. Molecular alteration of the *TP53* tumor suppressor gene is the most frequently occurring genetic event in serous carcinoma. p53 stabilization was demonstrated in SEIC, and now, *TP53* mutations and/or p53 stabilization is regarded as a crucial early event in serous carcinoma tumorigenesis [22]. Significance of *TP53* mutations and/or p53 stabilization in carcinogenesis from precursor lesions to type II ECs has also been demonstrated in an *in vivo* mouse model with endometrium-specific deletion of *Trp53* [23].

Dysregulation of the HER2/neu (ERBB2) receptor tyrosine kinase is another noteworthy molecular aberration in serous EC considering that HER2 is a proven druggable molecular target in other cancers, such as breast [24] or gastric cancer [25]. HER2 overexpression has been reported at a higher frequency up to 70% in serous ECs comparing to other histologic subtypes of ECs. The proportion of HER2 overexpression ranged from 1% to 47% in endometrioid ECs, although clinical significance has yet to be elucidated. Clear cell ECs have also been shown to overexpress HER2, but only a low number of cases were included for the analyses [26]. Other studied genetic aberrations that are implicated in serous carcinogenesis are somatic mutations in several genes, *FBXW7* [27, 28], *PPP2R1A* [27, 29], *STOP* [30], *CHD4* [31], *TAF1* [28], *PIK3CA* [32], and *PTEN* [27, 28], although functional effects of these molecular events remain to be elucidated [31, 33].

### 2.3. Clear Cell EC

Few studies on clear cell EC molecular composition have been reported. Molecular aberrations in *PIK3CA*, *PTEN*, *PPP2R1A*, *STOP*, *ARID1A*, *TAF1*, and *TP53* have been frequently reported genetic events in nonendometrioid ECs, including clear cell ECs [34–37]. In a recent study, an effort to overcome histopathologic misclassification resulting from interobserver variability was made, and mutations in selected genes were explored after a consensus on the diagnosis was achieved as the clear cell EC. Mutations in *PIK3CA*, *KRAS*, and *PIK3R1* genes were found in reclassified clear cell ECs [38]. Although whole exome sequencing of clear cell ECs was conducted in a recent study [36], only small numbers were analyzed, and comprehensive molecular characterization in clear cell ECs should be determined to clarify the exact molecular events in clear cell carcinogenesis.

### 2.4. Carcinosarcoma

Frequently found somatic mutations in uterine carcinosarcomas were present in *TP53* and genes involved in the PI3K/Akt/mTOR pathway, such as *PIK3CA*, *PTEN*, and *PIK3R1*. Other significantly but less frequently mutated genes were *FBXW7*, *PPP2R1A*, *KRAS*, and *ARID1A* [37, 38]. Based on the results of molecular analyses, such as genetic and immunohistochemical profiles, uterine carcinosarcoma has been considered to more closely resemble serous EC, rather than endometrioid EC [7, 39, 40]. However, cooccurrence of mutations in *TP53* and *PTEN* genes in tumors from carcinosarcoma, otherwise those two genes were mutually exclusive and showed a tendency to be found exclusively in one of two (endometrioid or serous) histologic subtypes in previous studies, suggested that carcinosarcoma originated from a common origin for carcinomatous and sarcomatous components [7, 39–41]. Analysis of somatic copy-number alterations revealed that most tumors were aneuploid. A high percentage of uterine carcinosarcomas had been shown to undergo a whole-genome-doubling event, a distinguishing feature from other tumors. Other potentially druggable molecular aberrations include a *POLE* gene mutation, a molecular event reported to have a favorable prognosis in patients with endometrioid ECs, and mutations in *AKT2*, *STK11*, *CCND1*, *CDKN2B*, *ERBB2*, *ERBB3*, *BRCA2*, *ATM*, *FGFR2*, and *SMARCA4* [7, 40, 41]. Defective DNA mismatch repair (MMR) has also been reported to be an early molecular event, giving rise to expectation for application of immune checkpoint inhibitors in the treatment of uterine carcinosarcomas with microsatellite instability (MSI) [40–42]. Potential of an immune checkpoint inhibitor as a promising targeted agent was also shown in a uterine carcinosarcoma patient harboring *POLE* mutation [43], reported to occur in about 2–4% of uterine carcinosarcomas [39–41].

### 2.5. Mismatch Repair Deficiency and Defect Replication Repair

Individuals with hereditary predisposition to Lynch syndrome (hereditary nonpolyposis colorectal cancer) harbor germline mutations in one of the following DNA MMR genes: *MLH1*, *MSH2*, *MSH6*, *PMS2*, or *EPCAM*. EC is one of the most frequently occurring extracolonic malignancies in patients with Lynch syndrome, and about up to 5% of ECs are caused by hereditary genetic aberrations [44, 45]. On the other hand, sporadic ECs with MMR defects are mainly attributed to methylation of the *MLH1* promoter, leading to epigenetic silencing. Microsatellite instability (MSI) is the phenotype of tumors with MMR defects resulting from hereditary or sporadic mutations of MMR genes. Loss of MMR has been reported to occur up to approximately 30% in endometrioid ECs, most frequently among histologic subtypes of ECs [46–49]. Defective MMR has been reported to occur less frequently in uterine carcinosarcomas than in endometrioid ECs, with a rate range of 3.5 to 21% [41, 42, 50] and reported to be uncommon in serous ECs [51]. In addition to MMR defects, somatic mutation of the exonuclease domain (ED) of the *POLE* gene, encoding a DNA polymerase epsilon catalytic subunit contributing to DNA replication and repair, results in genomic instability with neoantigen accumulation [33, 40, 45].

In contrast to a favorable prognosis of colorectal cancer patients with MMR defects, adverse clinicopathologic features such as higher-grade cancers and more frequent lymphovascular invasion were reported to be associated with MMR defects in endometrioid ECs. Furthermore, progression-free survival (PFS) of patients with tumors harboring epigenetic MMR defects was worse comparing with patients with proficient MMR endometrioid ECs [52]. On the other hand, *POLE*-ED mutation has been reported to be associated with favorable clinical outcomes in grade 3 endometrioid ECs [53, 54].

Regardless of relativeness to prognosis, defects in MMR or replication repair result in genomic instability leading to accumulation of neoantigen loads, rendering expectation for promising outcomes with the use of immune checkpoint inhibitors [45, 55].

### 2.6. Classification of EC according to Molecular Characterization

Integrated genomic analyses classified endometrioid and serous ECs into 4 categories according to a combination of somatic nucleotide substitutions, MSI, and somatic copy number alterations: *POLE*-mutated, MSI hypermutated, copy-number low/microsatellite stable (MSS), and copy-number high subgroups [33]. The *POLE*-mutated (ultramutated) group regarded tumors characterized by an increased frequency of C→A transversions, recurrent mutations at *POLE*^P286R^ and *POLE*^V411L^. Tumors in this group show exceedingly high mutation rates and an improved PFS. A clinicopathological review of *POLE*-ED-mutated ECs reported that these tumors were commonly high grade and presented endometrioid differentiation with obvious lymphocyte infiltrates [56]. The hypermutated subgroup included tumors with MSI, most with *MLH1* promoter hypermethylation, and with few somatic copy number alterations. The third group, tumors with low copy-number, most with MSS, was shown to have frequent mutations in *CTNNB1*, the only mutated gene occurring more frequently than in the MSI subgroup. Increased expression of progesterone receptor (PR) was found in this group. The copy-number high group consisted of serous ECs and a quarter of high-grade endometrioid ECs. Tumors in this group had frequent *TP53* mutations, few DNA methylation changes, and low estrogen receptor (ER)/PR levels [33].

## 3. Molecular Targeted Therapies

### 3.1. Hormonal Therapy

Although systemic chemotherapy is regarded as the preferred treatment modality, a subset of EC patients benefits from hormonal therapy. Low-grade metastatic or recurrent endometrioid ECs with positive ER have been shown to have a good response tendency to hormonal therapy [57, 58]. Efficacy of a variety of combination hormonal therapies, antiprogestin therapy, and antiestrogen monotherapy has been examined. The range of response rate (RR) of combination hormone therapies with tamoxifen and progestational agents (megestrol acetate, and medroxyprogesterone acetate) has been reported to be 19–33%, especially favorable for histologic grade I tumors, but a few patients have been reported to experience grade 4 thromboembolic events, including pulmonary embolism [59–61]. Results of other studies examining the efficacy of combination therapies with mTOR inhibitors and hormonal agents have also been reported. One study investigating RR and toxicities of temsirolimus combined with megestrol acetate and tamoxifen reported disappointing results with an RR of 14% accompanying an excess of thromboembolism [62]. On the other hand, another phase II clinical trial reported a positive result on a combination therapy with everolimus and an AI, letrozole, by showing an RR of 32% and tolerable toxicities in patients with recurrent endometrioid ECs [63]. Favorable response rates (RRs) up to 25% have also provided a basis for treatment with progestin monotherapy, such as megestrol acetate or medroxyprogesterone acetate in patients with advanced or recurrent ECs, particularly those with low-grade and positive ER/PR [64, 65]. Antiestrogen monotherapies with aromatase inhibitors, anastrozole [66] or letrozole [67], have shown low RRs in phase II clinical trials ranging from 9 to 10% in patients with advanced or recurrent ECs. In the study with letrozole, the expression rate was also evaluated for the following biomarkers: PR, ER, PTEN, phosphorylated PKB/Akt, BCL-2, p53, and HER2, but none of these correlated with the response to letrozole [67]. A phase II clinical trial on the efficacy of exemestane resulted in an RR of 10% and a lack of progression after 6 months in 35% of ER-positive patients with advanced or recurrent ECs [68]. Results on tamoxifen monotherapy have shown a modest activity with RRs from 10% to 20% in patients with advanced or recurrent ECs [69, 70]. Another selective estrogen receptor modulator, arzoxifene, showed an RR of 31% in patients with progestagen-sensitive, ER/PR-positive, advanced, or recurrent ECs [71]. Phase II clinical trials to examine the efficacy of a SERD, fulvestrant, have shown RRs of up to 16% in patients with ER-positive, advanced, or recurrent ECs [72, 73]. Results of clinical trials for hormonal therapies are summarized in Table 1.

### 3.2. Immune Checkpoint Inhibitors

Immunotherapies with checkpoint inhibitors have shown efficacy in solid tumors with MSI-high/defective MMR or with a high concentration of tumor-infiltrating lymphocytes (TIL) [7]. The expanded approval by FDA of the antiprogrammed cell death 1 (PD-1) antibody, pembrolizumab, to include treatment of MSI-high/defective MMR solid tumors refractory to prior treatment in 2017 has brought expectation to improve treatment outcomes of ECs, given that up to approximately 30% of endometrioid ECs have been reported to have MSI [46–49], and 48~100% of the tumors express PD-ligand 1 (PDL1) or PDL2 [74, 75].

Two phase II clinical trials investigating the efficacy of PD-1 blockade in MSI-high/defective MMR tumors refractory to prior therapy including ECs have found that pembrolizumab is highly effective in MMR-deficient cancers in terms of RR, PFS, and overall survival [76, 77]. Among 15 patients with MMR-deficient ECs, RR was reported to be 52% when treated with pembrolizumab in the phase II clinical trial [77]. The KEYNOTE-028 study was a phase Ib clinical trial evaluating the safety and efficacy of pembrolizumab in patients with locally advanced or metastatic PDL1-positive EC progressed to a previous standard therapy. A durable response was observed in 3 patients (13%) with a partial response among 24 patients with PDL1-positive EC [74]. RR was reported in up to 29% in patients with EC whose biomarker was unrevealed when treated with epacadostat, 2,3-dioxygenase 1 (IDO1) enzyme, combined with pembrolizumab in another phase I/II clinical trial (ECHO-202/KEYNOTE-037) [78]. Results of a phase Ib/II clinical trial examining another combination therapy with lenvatinib, a multikinase inhibitor, and pembrolizumab in patients with advanced and recurrent ECs have also reported its efficacy by showing 48% of RR with tolerable toxicities [79]. Efficacy of atezolizumab treatment, an anti-PDL1 antibody, has also been evaluated in patients with advanced ECs, and RR was reported to be 13% in a phase Ia clinical trial [80]. Despite favorable treatment outcomes of PD-1 blockade in EC patients, no benefit in a fraction of patients has been an issue to be solved by exploring definitive biomarkers for response. Several studies have suggested hypermutation or high TILs combined with positive immune checkpoint-related protein expression, as a result of MMR deficiency or *POLE* mutation, as possible biomarkers for response in treatment with immune checkpoint inhibitors [74, 80–82]. Results of clinical trials with immune checkpoint inhibitors are summarized in Table 2.

### 3.3. Agents Targeting PI3K/Akt/mTOR Signaling Pathway

Activation of the PI3K/Akt/mTOR pathway is known to be one of the crucial drivers to carcinogenesis of endometrioid ECs. As a matter of fact, somatic mutations of *PTEN* and *PIK3CA* are some of the most frequently occurring genetic aberrations in ECs [7, 15]. Treatment outcomes with single agents of mTOR inhibitors have shown a modest activity in patients with advanced or recurrent EC. Two phase II clinical trials evaluated the efficacy of mTOR inhibitors, temsirolimus and everolimus, in patients with ECs. Temsirolimus showed a superior RR (14%) in chemotherapy-naïve patients to RR (4%) of chemotherapy-treated patient with advanced or recurrent ECs [83]. No patients responded to everolimus treatment, another mTOR inhibitor, in patients with recurrent ECs [84]. Addition of temsirolimus on standard chemotherapy with paclitaxel and carboplatin did not show any benefit in terms of RR and PFS, compared to standard chemotherapy [85]. On the other hand, a randomized phase II clinical trial (GOG 3007), which has been reported only as an abstract form, comparing the efficacy of two combination therapies between everolimus/letrozole and hormonal therapy (medroxyprogesterone acetate/tamoxifen) in patients with advanced or recurrent ECs, undergoing on the basis of encouraging preliminary results [63], reported RRs of 53% and 43%, respectively, in chemotherapy-naïve patients [5].

### 3.4. The Agent Targeting ERBB2/HER2

Although amplification/overexpression of HER2 has been reported to be frequently found in ECs, particularly in serous ECs [26, 86], the benefits of anti-HER2 therapy have yet to be demonstrated. Treatment with single-agent trastuzumab in patients with advanced or recurrent ECs has shown a lack of efficacy in a phase II clinical trial. In addition, no association was shown between *HER2* amplification or overexpression and treatment outcomes [87]. Lapatinib, a small molecule inhibitor of epidermal growth factor receptor (EGFR) and HER2 receptor, showed limited activity in unselected patients with persistent/recurrent ECs, in a phase II clinical trial [88]. However, another recent randomized phase II clinical trial examining the efficacy of carboplatin and paclitaxel, a standard frontline chemotherapy in advanced ECs, with or without trastuzumab, reported encouraging results that PFS was improved in the trastuzumab group [hazard ratio, 0.44; 90% confidence interval (CI), 0.26-0.76; *p* = 0.005] in patients with advanced or recurrent HER2-positive serous EC [89]. An earlier study with two cases of EC harboring amplification of *HER2* also showed clinical responses when treated in combination with chemotherapy [90], giving rise to the significance of patients' selection, according to histological/molecular classification. The main resistance mechanisms to the anti-HER2 therapy suggested in serous ECs are overgrowth of HER2-negative cells in HER2 heterogeneous tumors, HER2 extracellular domain shedding, activation of downstream molecules of the signaling pathway, or activation of alternative signaling pathways [91]. Combination therapies with agents targeting molecules involved in resistance mechanisms of anti-HER2 therapy could be another way to solve this resistance and improve the treatment outcomes of the anti-HER2 therapy.

### 3.5. PARP Inhibitors

ARID1A deficiency, resulting in impairment of HR DNA repair [20], has provided a potential for clinical utility of PARP inhibitors in ARID1A-deficient EC. Frequent mutations of *ARID1A* in EC [33] led to the design of a randomized phase II clinical trial comparing the efficacy of olaparib (a PARP inhibitor), cediranib (a small molecule inhibitor targeting VEGFR, PDGFR, and FGFR), or the combination of both agents in patients with metastatic/recurrent ECs (ClinicalTrials.gov NCT 03660826), and its results are awaited.

### 3.6. Antiangiogenic Agents

Bevacizumab, a monoclonal antibody against vascular endothelial growth factor-A (VEGF-A), has shown activity across cancers. Efficacy of the single-agent bevacizumab was examined in recurrent or persistent ECs in a phase II clinical trial. Results in this study, an RR of 13.5% and a median PFS of 4.2 months, encouraged further investigation on its efficacy in EC patients [92]. The efficacy of addition of bevacizumab on chemotherapy, paclitaxel and carboplatin, in patients with advanced or recurrent EC was then examined in a phase II clinical trial. Although accrual to the study was discontinued for the initiation of a national randomized phase II clinical trial, the RR (73%; CI: 45-91) and median PFS (18 months, CI: 11-25) of 15 enrolled patients were shown to be promising [93]. Based on these preliminary clinical data, a randomized phase II clinical trial (MITO END-2) examining the efficacy of carboplatin/paclitaxel with or without bevacizumab was conducted in patients with advanced or recurrent EC. No benefit in the increase of PFS (10.5 vs. 13.7 months; HR, 0.84; *p* = 0.43) was shown by adding bevacizumab in chemotherapy [94]. No benefit of adding bevacizumab in chemotherapy was shown regarding PFS improvement in another randomized phase II clinical trial examining the efficacy of paclitaxel/carboplatin/bevacizumab, paclitaxel/carboplatin/temsirolimus, and ixabepilone/carboplatin/bevacizumab in patients with advanced or recurrent ECs, either [85]. However, nonsignificant improvement of PFS in the bevacizumab group observed in the MITO END-2 trial gives rise to the necessity of further evaluation with a larger population and reliable biomarkers.

Results of clinical trials for targeted agents other than hormonal agents or immunotherapy are summarized in Table 3.

## 4. Conclusion

Tailored therapy according to the molecular characterization has been expected to be one of the promising treatment modalities to improve the outcomes across solid cancers, despite various challenging interpretations of genetic aberrations to directly guide treatments. Efforts to clarify the molecular features of EC have established distinct molecular classifications. Despite the extensive amounts of data on molecular features of ECs, clinical application of targeted agents has been limited. Currently, systemic chemotherapy is the only standard treatment strategy for advanced and recurrent ECs. Targeted therapies based on molecular features might be one of the solutions for unmet needs for the treatment of ECs. However, several limitations to be solved exist to establish targeted therapies as a standard therapy in EC treatment. First, due to the fact that considerable genetic aberrations are context-dependent according to tumors, thorough preclinical studies should be performed to address exact function of the genetic aberrations. Second, exploration of appropriate biomarkers for targeted agents should be continued to improve the treatment outcomes. Third, the clinical benefit of targeted agents should be demonstrated in large prospective clinical trials. Efforts to develop molecular targeted agents are expected to improve treatment outcomes, especially high-grade histology subtypes, by extending clinically applicable treatment modalities.

## Figures and Tables

**Table 1:** Hormonal therapy in advanced or recurrent endometrial cancer.

Study	Design	No. of patients	Treatment	Primary end point	Results
*Monotherapy*					
*Antiestrogen therapy*					
Quinn and Campbell [69]	Case series	49	Tamoxifen 40 mg	RR	RR 20% Median survival of responder 34 mths
Thigpen et al. [70]	Prospective	68	Tamoxifen 40 mg	RR	RR 10% (90% CI 5.7-17.9) Median PFS 1.9 mths (90% CI 7-10.1)
McMeekin et al. [71]	Phase 2, open label	29	Arzoxifene 20 mg	RR	RR 31% (CI 25-51) Median PFS 3.7 mths (CI 1.9-6.6)
Emons et al. [73]	Phase 2	35	Fulvestrant 250 mg	RR	RR 11.4% TTP 2.3 mths (95% CI 2.5-6.6)
Covens et al. [72]	Phase 2	53	Fulvestrant 250 mg	RR	In ER-positive patients RR 16% Median PFS 10 mths Median OS 26 mths
Rose et al. [66]	Phase 2	23	Anastrozole 1 mg	RR	RR 9% (90% CI 3-23)
Ma et al. [67]	Phase 2	32	Letrozole 2.5 mg	RR	RR 9.4% (95% CI 2-25)
Lindemann et al. [68]	Phase 2, open label	51	Exemestane 25 mg	RR	In ER-positive patients RR 10% Median PFS 3.8 mths (95% CI 0.7-6.9)
					Median OS 13.3 mths (95% CI 7.7-18.9)

*Progestin therapy*					
Thigpen et al. [64]	Prospective	299	MPA 200 mg vs. MPA 1 g	RR	Low-dose group RR 25% Median PFS 3.2 mths Median OS 11.1 mths
					High-dose group RR 15% Median PFS 2.5 mths Median OS 7 mths
Lentz et al. [65]	Phase 2	54	MA 800 mg	RR	RR 24% Median PFS, 2.5 mths Median OS 7.6 mths

*Combination therapy*					
*Tamoxifen/progestational agents*					
Pandya et al. [59]	Phase 2, randomized	42	MA 320 mg vs. tamoxifen 20 mg & MA 160 mg	RR survival	MA group RR 20% Median OS 12 mths Tamoxifen & MA group RR 19% Median OS 8.6 mths
Whitney et al. [60]	Phase 2	58	Tamoxifen 40 mg & MPA 200 mg/day/alternating weekly	RR	RR 33% (95% CI 21-46) Median PFS 3 mths Median OS 13 mths
Fiorica et al. [61]	Phase 2	56	MA 160 mg altering with tamoxifen 40 mg	RR	RR 27% (90% CI 17-38)

*Hormonal agents/mTOR inhibitor*					
Fleming et al. [62]	Phase 2, randomized	71	Temsirolimus 25 mg vs. Temsirolimus 25 mg & MA 160 mg altering with tamoxifen 40 mg	RR	Temsirolimus group RR 22%
					Combination group RR 14%
Slomovitz et al. [63]	Phase 2	35	Everolimus 10 mg & letrozole 2.5 mg	CBR	CBR 40% RR 32%

No.: number; RR: response rate; mths: months; CI: confidence interval; PFS: progression-free survival; TTP: time to progression; ER: estrogen receptor; OS: overall survival; MPA: medroxyprogesterone acetate; MA: megestrol acetate; CBR: clinical benefit rate (complete response+partial response+stable disease ≥ 16 weeks).

**Table 2:** Immune checkpoint inhibitors in advanced or recurrent endometrial cancer.

Study	Design	No. of patients	Treatment	Primary end point	Results
KEYNOTE-28 [74]	Phase 1b	24	Pembrolizumab 10 mg/kg q 2 wks	RR	13% (95% CI 2.8-33.6)
ECHO-202/KEYNOTE-037	Phase 1/2, open label	7	Epacadostat 200 mg & pembrolizumab 200 mg	RR	RR 29%
Makker et al. [79]	Phase 1b/2, open label	23	Lenvatinib 20 mg & pembrolizumab 200 mg	RR	RR 48%
Fleming et al. [80]	Phase 1a	15	Atezolizumab 1200 mg or 15 mg/kg	Safety	RR 13% Median PFS 1.7 mths
					Median OS 9.6 mths

No.: number; RR: response rate; CI: confidence interval; PFS: progression-free survival; mths: months; OS: overall survival.

**Table 3:** Targeted agents in advanced or recurrent endometrial cancer.

Study	Design	No. of patients	Treatment	Primary end point	Results	*p*
*mTOR inhibitors*						
Oza et al. [83]	Phase 2	60	Temsirolimus 25 mg	RR	CTx-naïve group; RR 14% CTx-treated group; 4%	
Slomovitz et al. [84]	Phase 2, open label	35	Everolimus 10 mg	CBR	CBR 21% RR, none	
GOG-86P [85]	Phase 2, randomized	349	PC & bevacizumab vs. PC & temsirolimus vs. ixabepilone & carboplatin & bevacizumab	PFS	HR 0.81, 92% CI 0.63-1.02 HR 1.22, 92% CI 0.96-1.55 HR 0.87, 92% CI 0.68-1.11	>0.039
GOG 3007	Phase 2, randomized, open label, noncomparable		EL vs. PT	RR	RR 24 vs. 22% PFS 6.4 vs. 3.8 mths OS 20 vs. 16.6 mths	
*Anti-HER2 therapy*						
Fleming et al. [87]	Phase 2	33	Trastuzumab 2 mg/kg	RR	RR, none	
Leslie et al. [88]	Phase 2, open label	30	Lapatinib 1500 mg	6 mths PFS	10%, 90% CI 2.3-23.9	
Fader et al. [89]	Phase 2, randomized	61	PC vs. PC & trastuzumab	PFS	8 vs. 12.6 mths HR 0.44, 90% CI 0.26-0.76	0.005

*Antiangiogenic therapy*						
Aghajanian et al. [92]	Phase 2	52	Bevacizumab 15 mg/kg	6 mths PFS RR	6 mths PFS 40.4% RR 13.5%	
					Median PFS 4.2 mths Median OS 10.5 mths	
Simpkins et al. [93]	Phase 2	15	PC & bevacizumab	6 mths PFS	93%, 95% CI 82-100 Median PFS 18 mths (CI 11-25)	
MITO END-2 [94]	Phase 2, randomized	108	PC vs. PC & bevacizumab	PFS	PFS 10.5 vs. 13.7 mths, HR 0.84 RR 53.1 vs. 74.4% OS 29.7 vs. 40 mths, HR 0.71	0.43 0.24

No.: number; RR: response rate; CTx: chemotherapy; CBR: clinical benefit rate (complete response or partial response or stable disease ≥ 8 weeks); PC: paclitaxel+carboplatin; PFS: progression-free survival; HR: hazard ratio; CI: confidence interval; EL: everolimus 10 mg + letrozole 2.5 mg; PT: tamoxifen 40 mg + medroxyprogesterone acetate 200 mg; mths: months; OS: overall survival.
